# In Operando Imaging Electrostatic-Driven Disassembly and Reassembly of Collagen Nanostructures

**DOI:** 10.1021/acsnano.4c03839

**Published:** 2024-07-03

**Authors:** Clara Garcia-Sacristan, Victor G. Gisbert, Kevin Klein, Anđela Šarić, Ricardo Garcia

**Affiliations:** † Instituto de Ciencia de Materiales de Madrid, CSIC, c/Sor Juana Inés de la Cruz 3, 28049 Madrid, Spain; ‡ Institute of Science and Technology Austria, Klosterneuburg 3400, Austria; § Department of Physics and Astronomy, University College London, London WC1E 6BT, United Kingdom

**Keywords:** high-speed AFM, electrostatic, pH, amino acids, collagen

## Abstract

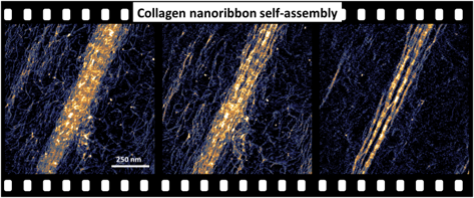

Collagen is the most abundant protein in tissue scaffolds in live organisms. Collagen can self-assemble in vitro, which has led to a number of biotechnological and biomedical applications. To understand the dominant factors that participate in the formation of collagen nanostructures, here we study in real time and with nanoscale resolution the disassembly and reassembly of collagens. We implement a high-speed force microscope, which provides in situ high spatiotemporal resolution images of collagen nanostructures under changing pH conditions. The disassembly and reassembly are dominated by the electrostatic interactions among amino-acid residues of different molecules. Acidic conditions favor disassembly by neutralizing negatively charged residues. The process sets a net repulsive force between collagen molecules. A neutral pH favors the presence of negative and positively charged residues along the collagen molecules, which promotes their electrostatic attraction. Molecular dynamics simulations reproduce the experimental behavior and validate the electrostatic-based model of the disassembly and reassembly processes.

Collagen is the main component in the extracellular matrix (ECM) of various tissues, including bone, tendon, cartilage, and cornea. It accounts for about 30% of protein content by weight in humans, which makes it the most abundant structural protein.^1−4^ Its abundance together with excellent biocompatibility and biodegradability properties underlines its relevance as a biomaterial^5−7^ in several applications from tissue engineering to bone regeneration.^7^

Collagens exhibit a hierarchy of structures in tissue scaffolds.^1−3,8^ Each collagen structure exhibits distinctive mechanical properties.^9,10^ Those properties are crucial to provide strength and elasticity to connective tissues such as tendons, ligaments, and bone. Collagen molecules can also self-assemble in vitro from solution to form nanofibrils and nanoribbons on the surface of some inorganic crystals.^11−15^

The physical and chemical features underlying the formation of collagen structures and scaffolds at the mesoscopic and macroscopic scales have been extensively studied.^1−10^ Those studies provide a solid understanding of the biological function of collagen structures. The atomic force microscope has provided high spatial resolution images and mechanical property maps of some collagen structures at the nanoscale.^15−23^ In contrast, much less is known on the interactions that promote and control the self-assembly of individual collagen molecules into higher order structures on inorganic crystals.^13−15^ The lack of knowledge is extensive in the description of the early stages of nanofibril formation. In vitro studies have shown that the pH of the solution is a key factor in collagen assembly;^12,24^ however, a detailed description of the physical processes governing the assembly of collagen molecules is missing. Those properties are relevant to develop collagen-based biocompatible materials and, more generally, to design protein–inorganic hybrid materials.^25−29^

The development of high-speed AFM (HS-AFM) has enabled the characterization in real time and with high spatial resolution of the dynamics of single proteins on surfaces.^30,31^ Those studies have generated direct approaches to imaging and understanding how biomolecules interact to function. More recently, HS-AFM has been applied to characterize the self-assembly of peptides and proteins.^15,30−35^ Here we characterize with high spatial and temporal resolutions the assembly and disassembly of individual collagen molecules. The measurements are performed with a high-speed AFM, which provides very high spatial and time resolutions, respectively, of 1 nm and 1 frame per second (0.1 μs per pixel) without disturbing the interaction among the collagen molecules. We reveal a transition from a collagen nanoribbon to a disordered distribution of collagen molecules on the mica surface by lowering the pH of the buffer solution from neutral to acidic conditions. By reversing the pH from acidic to neutral conditions, the initial nanoribbon structure is recovered. The reversibility of the process is very high. The experimental results are explained by introducing a coarse-grained molecular dynamics model. The model links the changes in the pH of the solution surrounding the collagen molecules and nanoribbons to the changes in surface charge distribution of the collagen molecules.

## Results and Discussion

### Collagen Nanostructures

**Figure 1:**
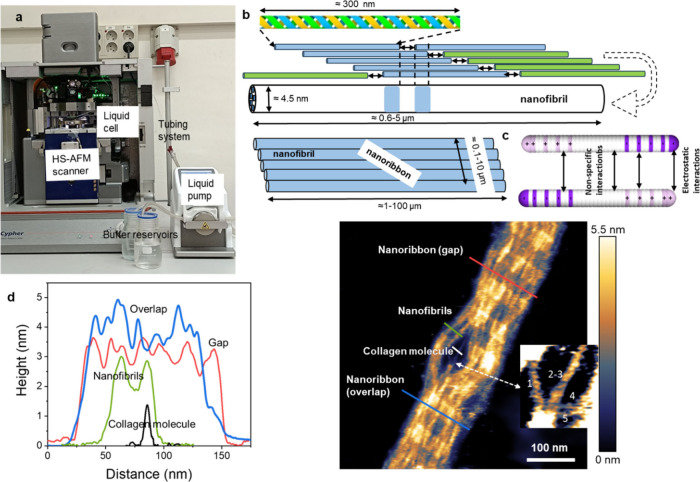
Elements of the HS-AFM setup and schemes of collagen nanostructures. (a) HS-AFM base and fluid cell, pump, and buffer reservoirs. (b) Scheme of a collagen molecule (triple helix), a collagen nanofibril, and a nanoribbon. The collagen molecule has a triple-helix structure. The collagen nanofibril is formed by the assembly of five collagen molecules. A collagen nanoribbon is formed by the assembly of several collagen nanofibrils. (c) Scheme of the collagen mimetic molecule and the electrostatic and nonspecific attractive interactions. Regions of negative and positive charges are plotted, respectively, in magenta and pink. (d) Height values of collagen molecules, nanofibrils, and nanoribbon (overlap and gap regions). The data were extracted from the accompanying AFM image. The inset (30 nm × 32 nm) shows five collagen molecules emerging from a nanofibril. The image was obtained in buffer by applying a peak force on the nanoribbons of 390 pN. AFM image of 1024 × 1024 pixels. AFM data: *f* = 451 kHz, *k* = 0.15 nN/nm, *Q* = 1.2; *A*_0_ = 2.55 nm and *A*_sp_ = 2.06 nm.

Figure 1 shows a scheme of the experimental setup and the schemes of the collagen structures relevant here. The key elements of the instrument are a high-speed AFM unit operated in an amplitude modulation mode (tapping mode),^36,37^ a fluid cell with inlets and outlets for exchanging the buffer, and a peristaltic pump to provide a quick and noise-free buffer exchange (Figure 1a). The experiment involves three structures from type I collagen, the triple helix collagen molecule, nanofibrils, and nanoribbons (Figure 1b). Following the model of Hulmes,^38^ we define a nanofibril as a collagen nanostructure made of five staggered collagen molecules. It is the smallest structure (in diameter) that preserves the characteristic D-band periodicity of observed native mesoscopic collagen fibrils. A nanoribbon is formed by the sideways arrangement of several nanofibrils.

To minimize collagen deformation during imaging, HS-AFM is performed by applying forces in the sub-nN range. Those forces are required to image proteins without introducing irreversible deformations.^39^ The forces applied in these experiments were in the 300–600 pN range. In tapping mode AFM, the forces cannot be measured directly while imaging.^36^ They were determined by using a dynamic AFM simulator (dForce 2.0).^40^

The height of the collagen nanostructures is determined from tapping mode AFM topographic images. Figure 1d shows a collagen system with the presence of three different nanostructures: collagen molecules, nanofibrils, and nanoribbons. This combination of collagen nanostructures in a single image allows for a direct and unbiased comparison of the height of the different nanostructures. The height values (average) for collagen molecules, nanofibrils, and nanoribbons are respectively 1.5 nm, 3 nm, and 3.5 nm (gap) and 4.5 nm (overlap). The high spatial resolution tapping mode AFM image indicates the threading of five collagen molecules into a nanofibril (inset). The image agrees with the model sketched in Figure 1a. That model establishes that a nanofibril is made of five collagen molecules. The image indicates that the structural model developed by X-ray diffraction from mesoscopic collagen fibrils^8,38^ applies also to the two-dimensional collagen nanostructures studied here.

### Real-Time Imaging Disassembly and Reassembly

**Figure 2:**
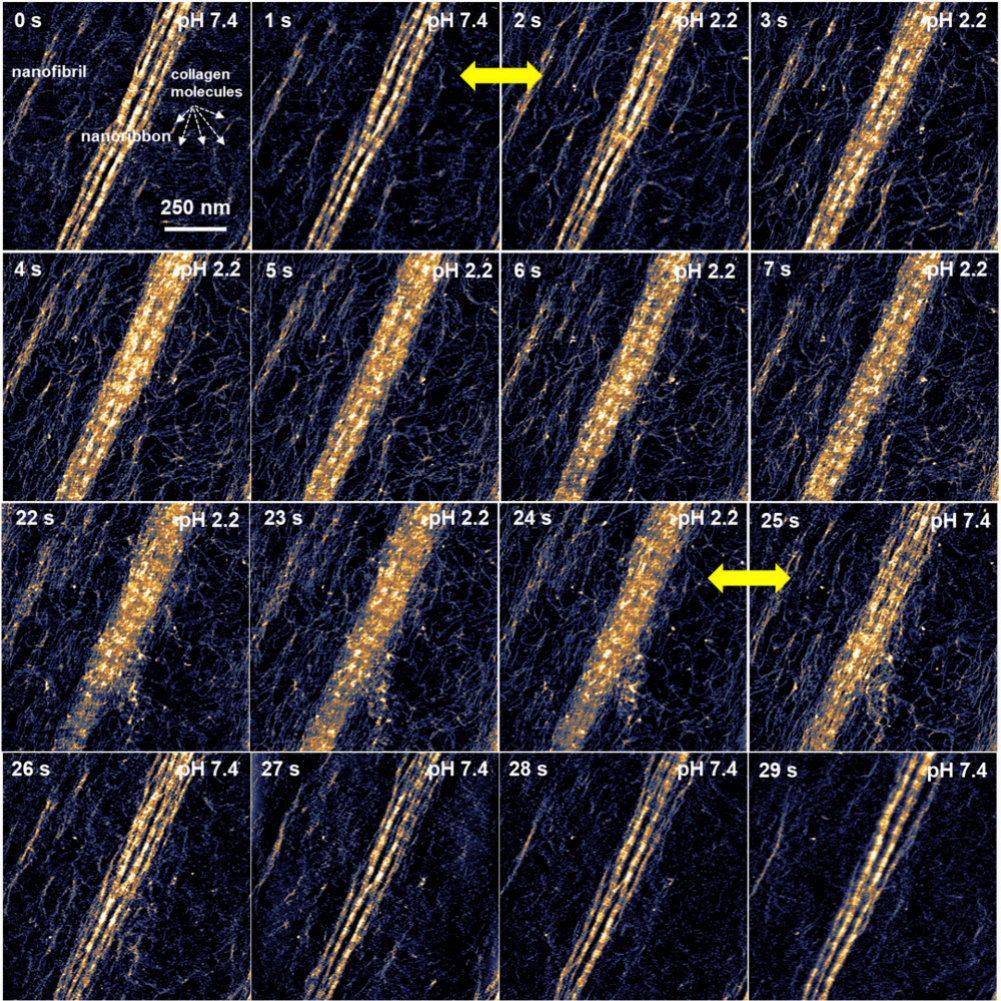
Real-time imaging disassembly and reassembly of collagen nanoribbons. Sequence of topography images showing the disassembly and reassembly of a collagen nanoribbon on a mica surface (see Movie S1 in Supporting Information). The disassembly is caused by lowering the pH of the solution from 7.4 (neutral) to 2.2 (acidic). The reassembly of the nanoribbon is activated by increasing the pH from 2.2 to 7.4. The frames involved in a pH transition (neutral to acidic or acidic to neutral) are marked by an arrow (in yellow). The images were obtained in buffer by applying a peak force on the nanoribbons of 390 pN. Imaging rate, 1 fps (512 × 256 pixels). Additional HS-AFM data: *f* = 522 kHz, *k* = 0.15 nN/nm, *Q* = 1.4; *A*_0_ = 3.5 nm and *A*_sp_ = 3.2 nm.

Figure 2 shows a sequence of in situ HS-AFM images of several nanofibrils and nanoribbons on a mica surface. The whole sequence of frames is found in Figure S1. The time-series image shows the disassembly and reassembly of collagen molecules as the pH of the buffer solution is changed from 7.4 (neutral) to 2.2 (acidic) and back to 7.4. We estimate that the time required to set a uniform value of pH in the fluid cell is less than 1 s. This estimation is based on the volume of the solution under the tip (2 μL) and the flow rate of the fluid (2 μL/s).

At time *t* = 0 s (pH = 7.4) the HS-AFM image shows the presence of several collagen molecules, a few scattered nanofibrils, and a collagen nanoribbon. The nanoribbon shows the characteristic stripe pattern (D-band) of in vivo collagen fibrils. The pattern alternates overlap and gap regions with a spatial periodicity of about 67 nm (Figure S3). That value agrees with the values reported for macroscopic type I collagen fibrils.^2,8,9,21^ The nanofibrils are oriented parallel to the main crystal directions of the mica lattice. The electrostatic interaction between the mica surface and the collagens controls the long-range alignment of the collagen nanofibrils and nanoribbon on the mica.^13,14^

The frames recorded from *t* = 1 to 24 s show the disassembly of the collagen nanoribbon and nanofibrils. The disassembly is activated by lowering the pH from 7.4 to 2.2. The AFM height cross sections indicate that disassembly proceeds by the removal of the collagen molecules located on the top of the nanoribbon (see below). This process blurs the overlap–gap pattern of the nanoribbon but does not lead to the total disassembly of the nanoribbon. We observe that the majority, if not all, of the collagen molecules remain attached to the mica surface near the nanoribbon. Electrostatic interactions between the negatively charged mica surface and the positively charged collagen molecules keep collagens attached to the mica. This feature will act as a memory effect that will facilitate the reassembly of the nanoribbon.

At *t* = 24.1 s the pH is increased to 7.4, which sets the reassembly of the nanoribbons (frames *t* = 25 to 29 s). No appreciable changes are observed after *t* = 27 s, which indicates that the reassembly happens in a time scale of 1–2 s.

**Figure 3:**
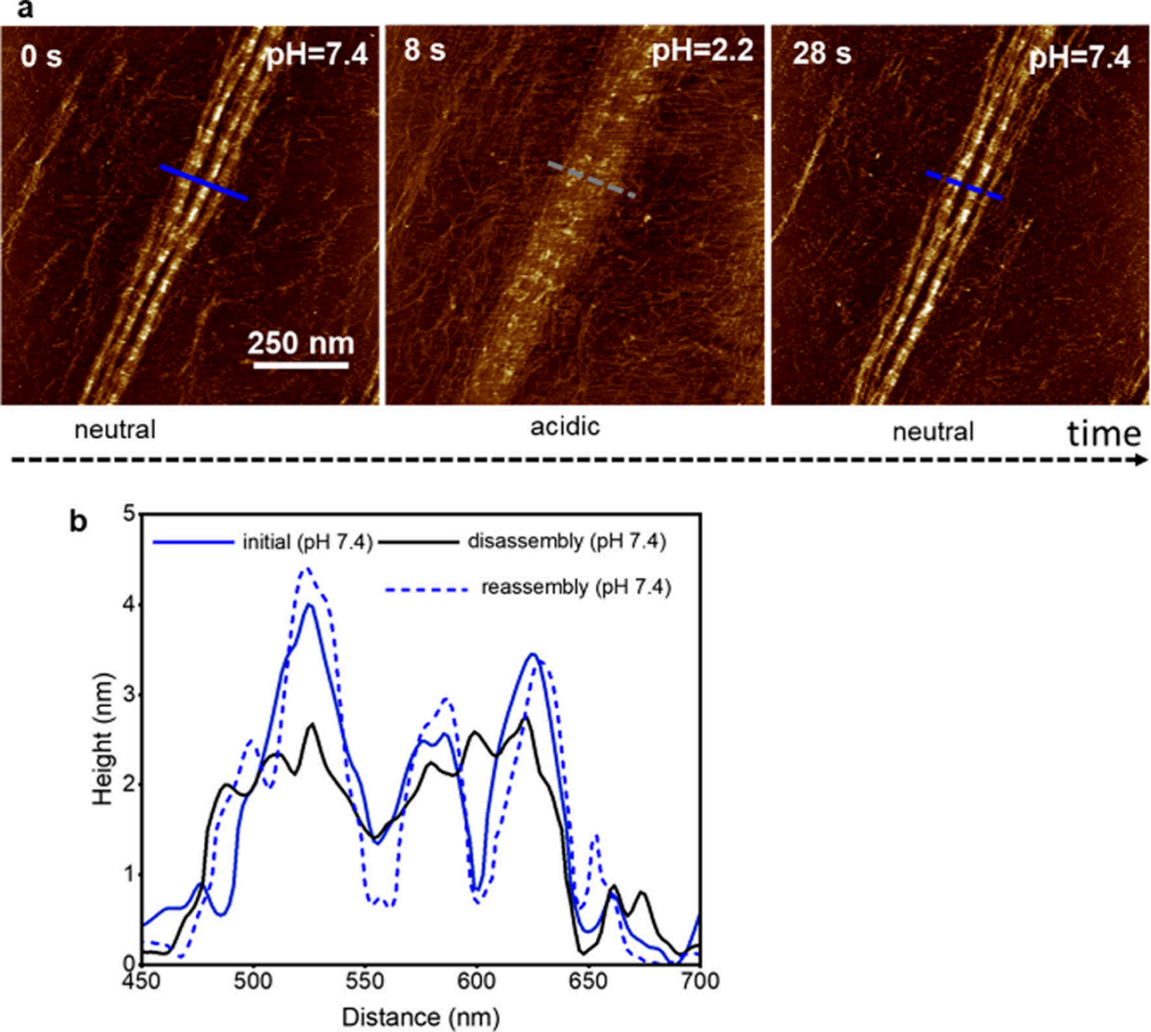
Comparison of initial and reassembled nanoribbons (experiment). (a) AFM height images of a nanoribbon under different pH conditions. (b) Height cross sections across the lines marked in the image. Dark blue (initial), black (pH = 2.2), light blue (reassembled nanoribbon at pH = 7.4). The images were obtained in buffer by applying a peak force of 390 pN. Imaging rate, 1 fps (512 × 256 pixels). Additional HS-AFM data: *f*_1_ = 522 kHz, *k*_1_ = 0.18 nN/nm, *Q*_1_ = 1.4; *A*_0_ = 3.5 nm, *A*_sp_ = 3.2 nm.

Figure 3a shows the AFM height images of a collagen nanoribbon before, during, and after completing a pH cycle. The images show that the initial and reassembled nanoribbon are very similar in both size and shape. For example, the bifurcation marked in the initial nanoribbon is reproduced in the reassembled nanoribbon. Furthermore, both nanoribbons have the same number of fingers. At the nanoscale, say 100 × 100 nm^2^ regions, it might be hard to spot differences between frames recorded at *t* = 0 s and *t* = 28 s. However, those differences show up by increasing the image range, which gives rise to substantially different collagen nanoribbons. Thermal energy fluctuations introduce some orientational changes in the nanofibrils. Those changes propagate and accumulate along the nanoribbon. For example, at the top of the image the nanoribbon marked by an arrow at *t* = 29 s diverges appreciably from its initial orientation at *t* = 0 s. Those fluctuations prevent the exact replication of the nanoribbon pattern before and after the disassembly process. Figure 3b compares the overlay of the height profiles (marked in Figure 3a). The height profiles of the initial and the reassembly nanoribbon are similar but they do not exactly match. The above results have been reproduced all the times that a similar experiment was performed (Figure S2).

**Figure 4:**
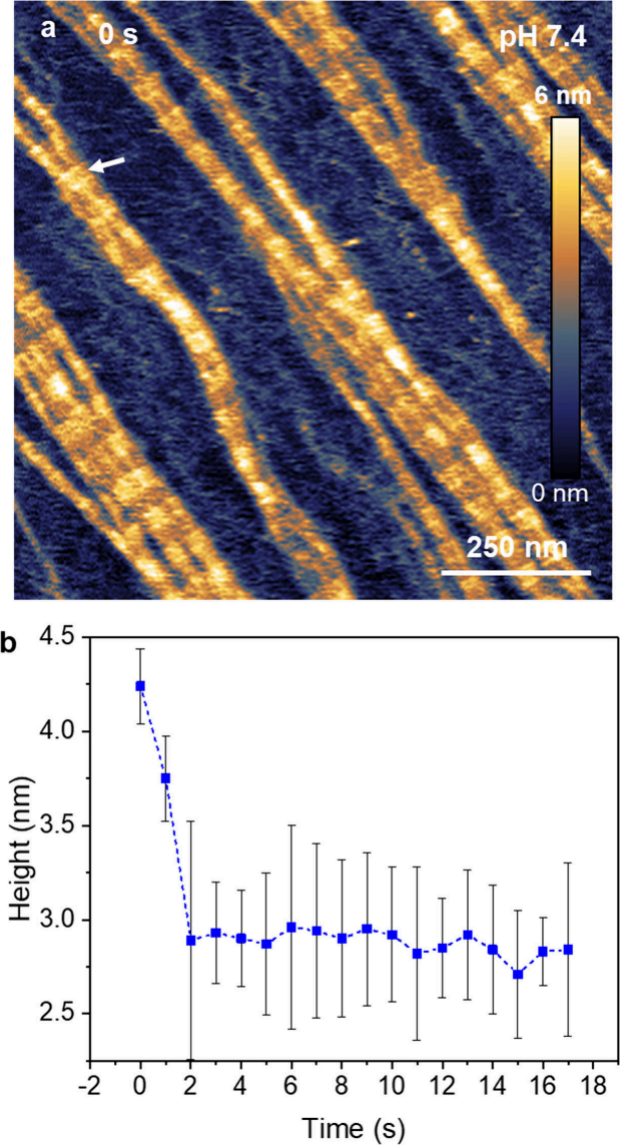
Height evolution during disassembly. (a) AFM topography images of collagen nanoribbons on mica (pH = 7.4). (b) Time evolution of the height of the location marked by an arrow in (a). At time *t* = 0.1 s, the pH was lowered to 4.25 and kept at that value during the duration of the measurements. The image was taken by applying a peak force of 610 pN. Imaging rate, 1 fps (512 × 256 pixels). Additional HS-AFM data: *f*_1_ = 528 kHz, *k*_1_ = 0.15 nN/nm, *Q*_1_ = 1.4; *A*_0_ = 3.5 nm, *A*_sp_ = 2.1 nm.

Quantitative details of the processes involved in the nanoribbon disassembly are obtained by plotting the nanoribbon height as a function of time. To that purpose, we choose a small section of a nanoribbon imaged at pH = 7.4 (Figure 4a) and follow its evolution at a lower of pH. Figure 4b shows that the nanoribbon height decreases by about 1.3 nm during the first 2 s of exposure to a pH = 4.3. From then onward (*t* = 2 to 14 s) the height remains practically stable at 3.2 nm. The height decrease in the first 2 s is very close to the nominal diameter of a collagen molecule (≈ 1.5 nm). This result indicates that the disassembly happens by the removal of the top collagen molecules forming the nanoribbon. Height changes below 0.3 nm might be associated with the difficulty to choose the same spot of the nanoribbon with sub-1 nm accuracy.

### Molecular Dynamics Simulations

To explore quantitatively the role played by the distribution of electric charges in the disassembly and reassembly of the nanoribbons, we apply coarse-grained molecular dynamics (MD) simulations. The simulations incorporate collagen-mimetic molecules, which are known to feature collagen-like self-assembly properties.^14,41^ In the simulations, the collagen-mimetic molecules are described as flexible rods that carry a sequence of charges (Figure 1c). The mimetic molecules are surrounded by an implicit water bath kept at 27 °C (300 K). The charges are located in some of the amino acid residues of the molecule. In the simulations, the collagen-mimetic molecules interact via screened electrostatic interactions as well as via generic nonspecific attractive interactions (Figure 1c). Depending on the interaction strengths, such rods might form either clusters or collagen-like nanoribbons and nanobundles.^40^ We model the electrostatic interactions using a Debye–Hueckel potential and Lennard-Jones potential for nonspecific interactions. To simulate the effect of a change in pH performed in the experiment, we vary the sequence of charges carried by the collagen-mimetic rods.

**Figure 5:**
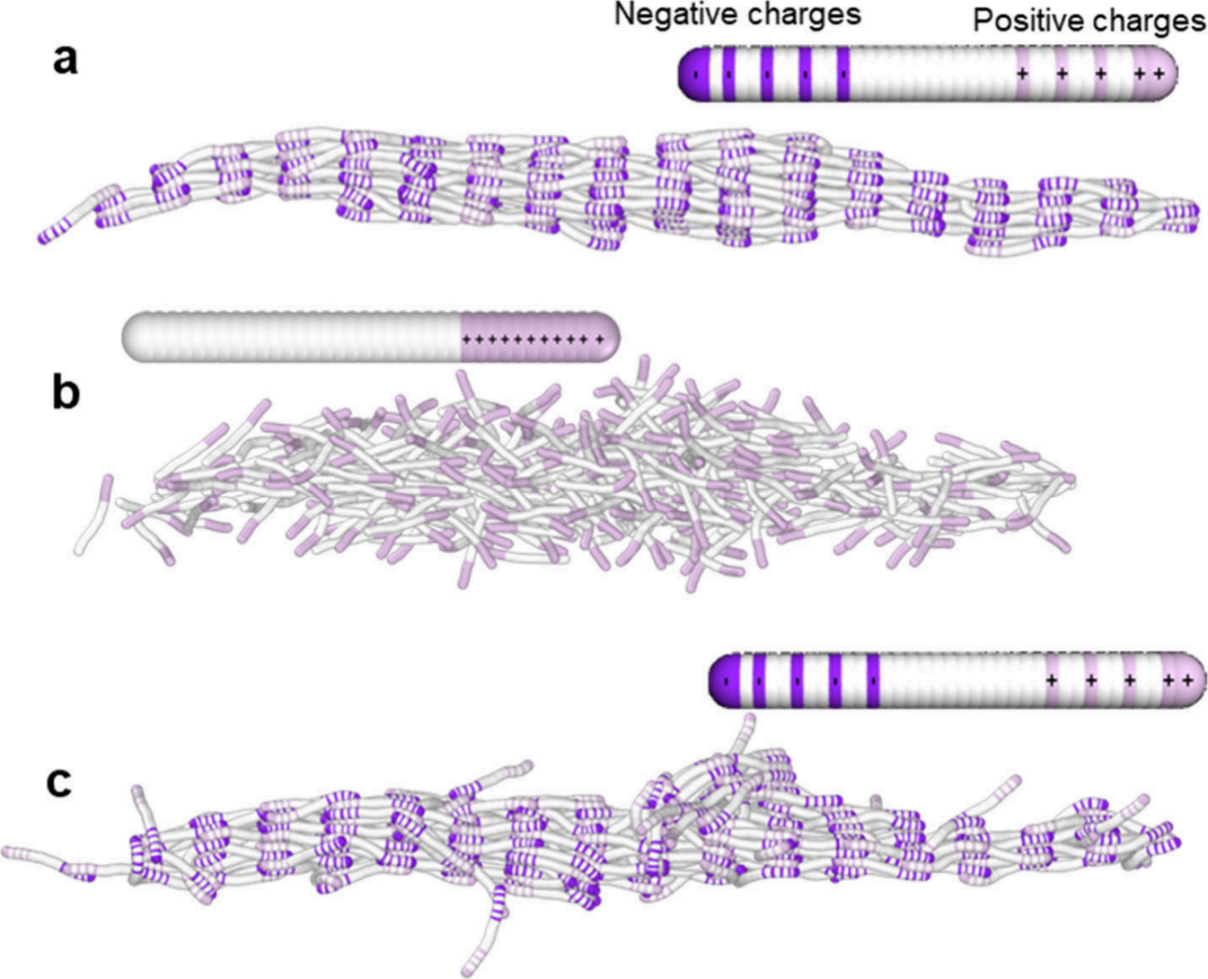
MD snapshots of a collagen-mimetic nanoribbon. (a) Nanoribbon for a charge distribution equivalent to pH = 7.4. (b) Disassembled nanoribbon obtained from (a) by removing the negative charges of the rod and adding additional positive charges as illustrated in the accompanying rod. (c) Reassembly of the nanoribbon. The nanoribbon reassembled from the configuration shown in (b) by reintroducing negative charges to the collagens. The rod model of the collagen accompanying the snapshots shows the actual charge distribution along the collagen molecule used in the different stages of the simulations. Regions of negative and positive charges are shown, respectively, in magenta and pink.

Figure 5 shows some snapshots of the simulations. Figure 5a shows a nanoribbon structure of the collagen molecules obtained by introducing a charge distribution equivalent to a pH = 7.4. Figure 5b shows the collagen structure obtained by lowering the pH from 7.4 to 2.2. Here, lowering the pH neutralizes negative charge residues while introducing additional positively charged residues, which, in turn, facilitates the disassembly of the collagen nanostructure. Figure 5c shows the collagen structure obtained by increasing the pH from 2.2 to 7.4. The nanoribbon structure is recovered. A movie showing the sequence of the simulations is found in the SI.

**Figure 6:**
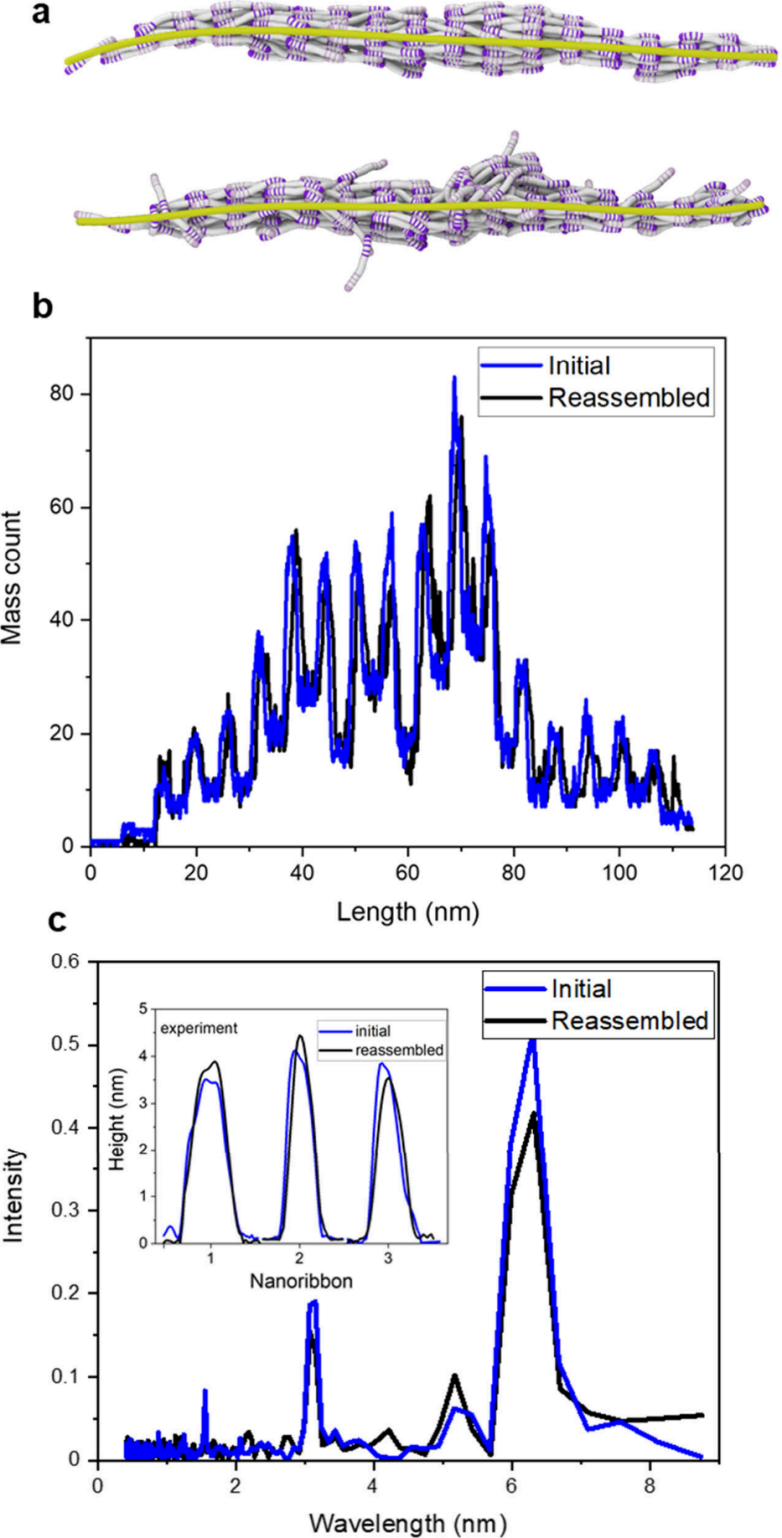
Comparison of initial and reassembled nanoribbons (simulation). (a) Top, initial nanoribbon structure. Bottom, final nanoribbon structure after disassembly and reassembly. (b) Mass distribution profile along the backbones of the nanoribbons. (c) FFT of the mass profiles shown in (b). The inset shows the height profiles (experiment) of three collagen nanoribbons before disassembly and after reassembly.

The MD simulations provide the mass distribution of the nanoribbons taken along the backbone axis (yellow splines in Figure 6a) before the disassembly and after the reassembly processes. The mass profiles (Figure 6b) and the fast Fourier transform (FFT) of the mass profiles (Figure 6c) of the initial and reassembly nanoribbon are similar. The inset (experiment) shows the height cross section of three collagen nanostructures before disassembly and after reassembly. The cross sections are proportional to the mass profiles. They enable a direct semiquantitative comparison between experiment and simulations. The simulations reproduce well the experimental behavior. The HS-AFM images used to obtain the above height cross sections are found in the SI (Figure S4).

The agreement obtained between simulations and experiments underline that a dynamic change in the collagen charge distribution is sufficient to drive the disassembly or reassembly of collagen nanostructures. For example, the reassembly starts by increasing the pH of the solution. As a consequence, the charge of some amino acid residues of the collagen molecules changes from neutral to negative. This process has two effects. First, it weakens the electrostatic interaction between the collagen and the mica surface. As a result, the collagen molecules are released from the mica surface. Second, the negatively charged residues of a collagen molecule exert an attractive force on the positively charged regions of neighboring collagen molecules, which eventually leads to the self-assembly of collagen nanofibrils and nanoribbons.

The role of attractive electrostatic interactions in stabilizing the structure of collagen nanoribbons might not apply to stabilize the folded state of a collagen molecule. Nuclear magnetic resonance spectroscopy experiments have underlined the effect of quinary interactions on modulating the electrostatic interactions within a protein.^42^

## Conclusions

We have observed in real time and with molecule-scale spatial resolution the disassembly and reassembly of collagen nanostructures including nanofibrils and nanoribbons. The disassembly and reassembly processes are controlled by the electrostatic interactions between the collagen molecules. The disassembly of the nanoribbons is initiated by lowering the pH of the buffer solution from neutral to acidic conditions. The in situ high-speed AFM images show that the disassembly happens by removing the collagen molecules located at the top of the nanoribbon. We show that upon reversing the pH of the solution, that is, by recovering the initial neutral pH, the nanoribbon reassembles to reestablish its initial shape. The experimental data are reproduced by performing molecular simulations using collagen-mimetic molecules. The agreement obtained between the simulations and the experiments indicates that the disassembly and reassembly processes are driven by repulsive and attractive electrostatic interactions between amino-acid residues along the molecule. The disassembly is initiated by converting negatively charged residues into neutral groups. The reassembly is initiated by transforming those neutral residues into negatively charged residues. The sign of the charge is controlled by the pH of the solution. These results demonstrate that the assembly, disassembly, and reassembly of collagen molecules, nanofibrils, and nanoribbons can be systematically and reversibly controlled by the pH of the solution. Acidic conditions activate the disassembly of the nanoribbons, while reestablishing a neutral pH sets the nanoribbon reassembly. The phenomena observed here might apply to other protein systems. Therefore, our findings offer a reliable and reproducible tool for designing protein–inorganic hybrid materials.

## Materials and Methods

### Collagen Preparation

Collagen molecules were obtained from monomeric bovine collagen type I (PureCol, CellSystems GmbH). Several phosphate buffer solutions (PBS) were prepared. The as-received PBS (pH = 7.4) contained 0.01 M phosphate, 0.0027 M potassium chloride, and 0.137 M sodium chloride. The concentration of KCl was increased to 300 mM to define the standard buffer in the experiments. From that buffer we prepared two acidic solutions at pH 2.2 and 4.3. The pH was reduced by adding HCl respectively at concentrations 1 mM and 0.1 mM. The chemicals were purchased from Sigma-Aldrich.

The imaging buffer consisted of PBS (Sigma-Aldrich) with 300 mM KCl (Sigma-Aldrich), pH 7.4, in a solution volume of 200 mL.

### High-Speed AFM Parameters and Measurements

A commercial HS-AFM platform and software (Cypher VRS, Oxford Instruments, USA) were used in these experiments. The HS-AFM was operated in liquid in the amplitude modulation AFM mode.^36,37^ Mechanical excitation was used to excite the vibration of the cantilever. The experiments were performed with very small cantilevers (7 μm × 2 μm × 80 nm) (USC-F1.2-k0.15, NanoAndMore, Germany). Typical values of the resonant frequencies, force constants, and quality factors in liquid were *f*_*1*_ ∼ 520 kHz, *k*_1_ ∼ 0.15 N/m, and *Q*_1_ ∼ 1.5. Typical values of the free amplitudes *A*_0_ and set-point *A*_sp_ amplitudes were respectively 3 nm and *A*_sp_ ∼ (0.7–0.9)*A*_*0*_. The images were recorded at 1 fps with 512 × 256 pixels unless otherwise stated.

The as-received collagen solution was diluted to a concentration of 3.0 μg mL^–1^ by using the neutral PBS buffer and rapidly injected into a freshly cleaved muscovite mica disk (Grade V-1, Alpha Biotech Ltd.) placed inside the fluid cell of the AFM. Imaging started without further delay. The buffer solutions were injected by using a digital peristaltic pump (Masterflex MFLX78001-70). They were injected with a flow rate of 0.002 mL/s. The temperature of the buffer in the cell was 27 °C.

## Supporting Information Available

Figures S1, S2: time series of disassembly and reassembly of a collagen nanoribbon on a mica surface; Figure S3: AFM image of a collagen nanoribbon and FFT of the rectangular region marked in the image; Figure S4: representative examples of the frames and height cross sections used to determine the height evolution during disassembly; additional details on MD simulations (PDF)
Movie S1: high-speed AFM movie of the disassembly and reassembly of collagen nanofibrils (AVI)

## Supplementary Material

nn4c03839_si_001.pdf

nn4c03839_si_002.avi
