# Supplementary material for: In Operando Imaging Electrostatic-Driven Disassembly and Reassembly of Collagen Nanostructures
Source: ACS Nano. 2024 Jul 3;18(28):18485–92. doi: 10.1021/acsnano.4c03839 (PMC11256892; doi:10.1021/acsnano.4c03839)
Supplement: Supplementary file 1 — nn4c03839_si_001.pdf [file nn4c03839_si_001.pdf]

Supporting Information

In operando imaging electrostatic-driven disassembly and reassembly of collagen nanostructures

Clara Garcia-Sacristan[†], Victor G. Gisbert[†], Kevin Klein[‡,•], Anđela Šarić[‡], Ricardo Garcia[†]*

[†]Instituto de Ciencia de Materiales de Madrid, CSIC, c/ Sor Juana Ines de la Cruz 3, 28049 Madrid, Spain

‡Institute of Science and Technology Austria, Klosterneuburg, Austria

•UCL, London, United Kingdom

* Email: r.garcia@csic.es.

List of contents

- **Movie captions (Movie S1)**
- **Figure S1. Time series of collagen nanostructures at different pH values (I).**
- **Figure S2. Time series of collagen nanostructures at different pH values (II).**
- **Figure S3. Periodicity D-band.**
- **Figure S4. Height variations during disassembly.**
- **Molecular dynamics simulations**

Movie Captions

Movie S1. High-speed AFM movie of the disassembly and reassembly of collagen nanofibrils. The images shown in Fig. 1 were extracted from the movie. The measurement was performed with a free amplitude A_{01} = 3.5 nm and A_{sp} = 3.2 nm. The maximum force F_{peak} exerted on the collagen was of 390 pN. The movie was obtained at 300 Hz, 1 µm scan size, 512x256 pixels and 1 fps. The cantilever parameters were f = 522 kHz, k = 0.15 nN/nm, Q = 1.4 (USC-F1.2-k0.15, NanoAndMore).

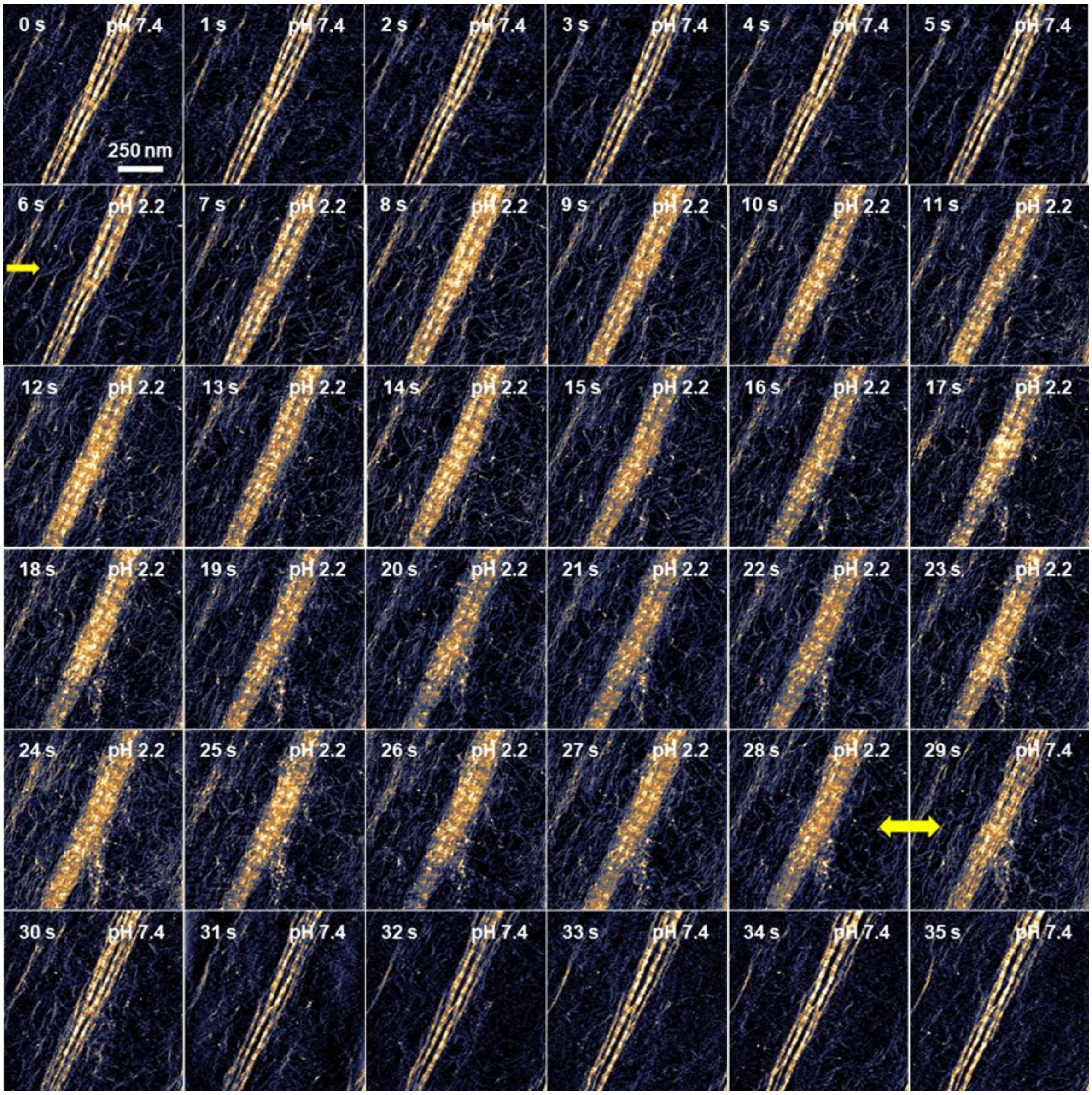

Supplementary Figure 1. Time series of disassembly and reassembly of a collagen nanoribbon on mica surface (see video S1). The nanoribbon was imaged continuously for 1 minute. The disassembly is caused by lowering the pH of the solution from 7.4 (neutral) to 2.2 (acidic). The reassembly of the nanoribbon is activated by increasing the pH from 2.2 to 7.4. The frames involved in a pH transition (neutral to acidic or acidic to neutral) are marked by an arrow (in yellow). The images were obtained in buffer by applying a peak force on the nanoribbons of 390 pN. Imaging rate, 1 fps (512x256 pixels). Additional HS-AFM data, f = 522 kHz, k = 0.15 nN/nm, Q = 1.4; A_0 = 3.5 nm and A_{sp} = 3.2 nm.

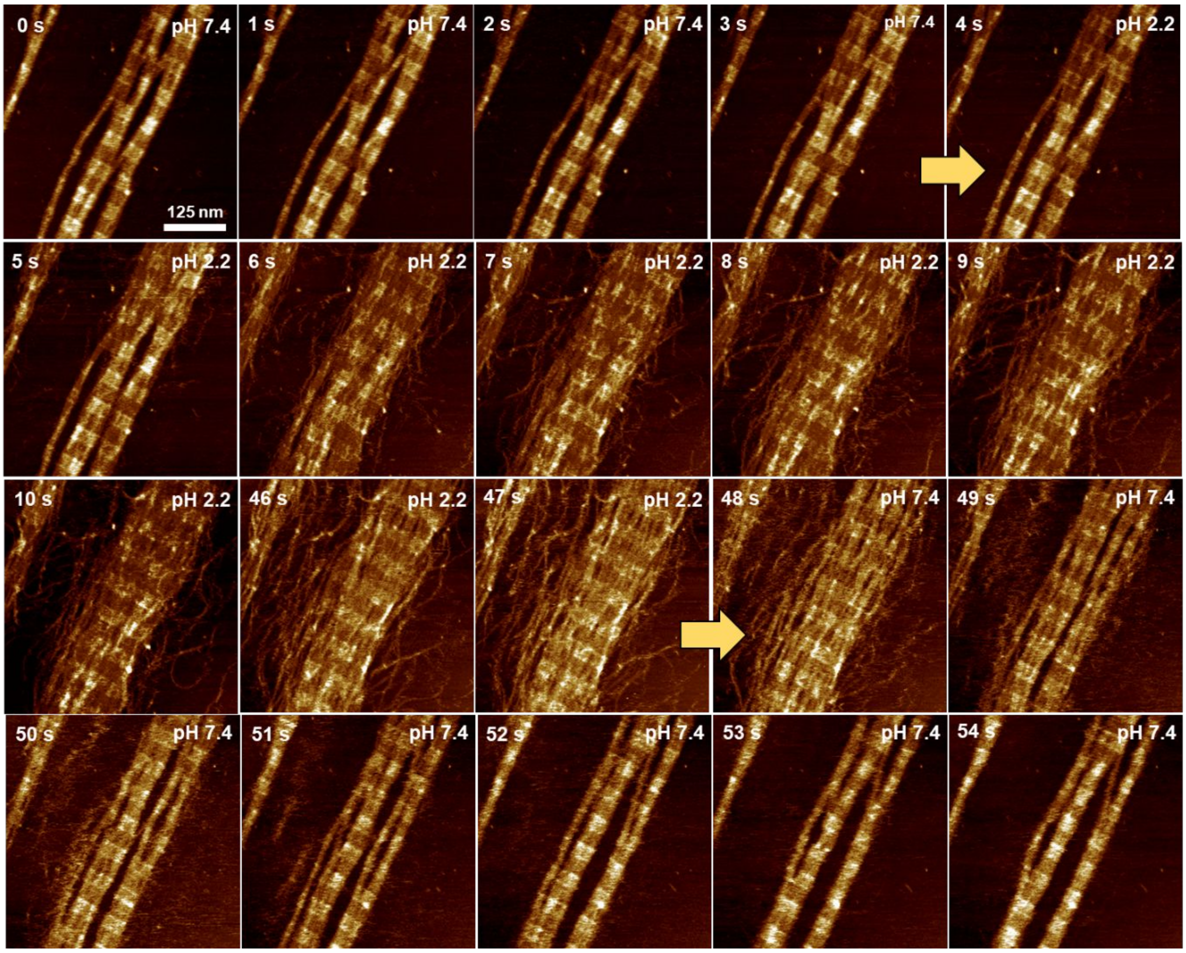

Supplementary Figure 2. Time series of disassembly and reassembly of a collagen nanoribbon on mica surface. The images replicate the findings showed in Figure 2 of the main text. The disassembly is caused by lowering the pH of the solution from 7.4 (neutral) to 2.2 (acidic). The reassembly of the nanoribbon is activated by increasing the pH from 2.2 to 7.4. The frames involved in a pH transition (neutral to acidic or acidic to neutral) are marked by an arrow (in yellow). The images were obtained in buffer by applying a peak force on the nanoribbons of 390 pN. Imaging rate, 1 fps (512x256 pixels). Additional HS-AFM data, f = 678 kHz, k = 0.15 nN/nm, Q = 1.7; A_0 = 3 nm and A_{sp} = 2.6 nm.

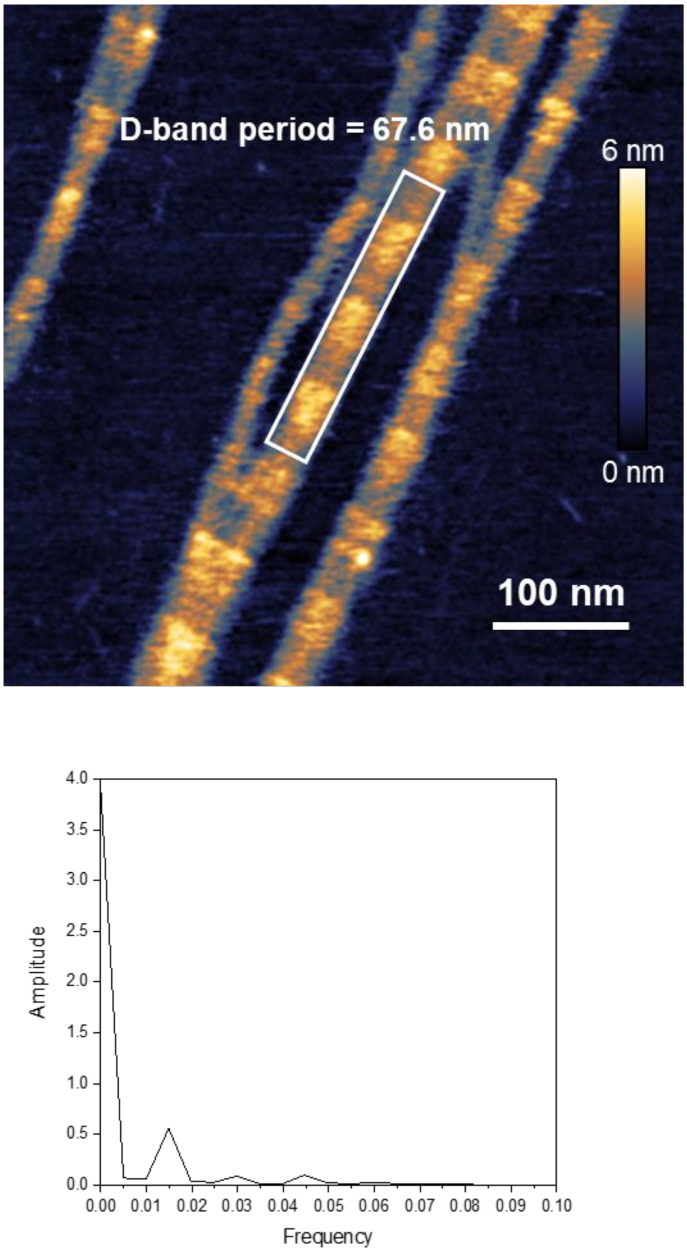

Supplementary Figure 3. AFM image of a collagen nanoribbon (top) and FFT of the rectangular region marked in the image. The spatial frequency is of 0.0149 which corresponds to a periodicity of 67.6 nm. Imaging rate, 1 fps (512x256 pixels). Additional HS-AFM data, f = 564 kHz, k = 0.15 nN/nm, Q = 1.8; A_0 = 2.4 nm and A_{sp} = 1.6 nm.

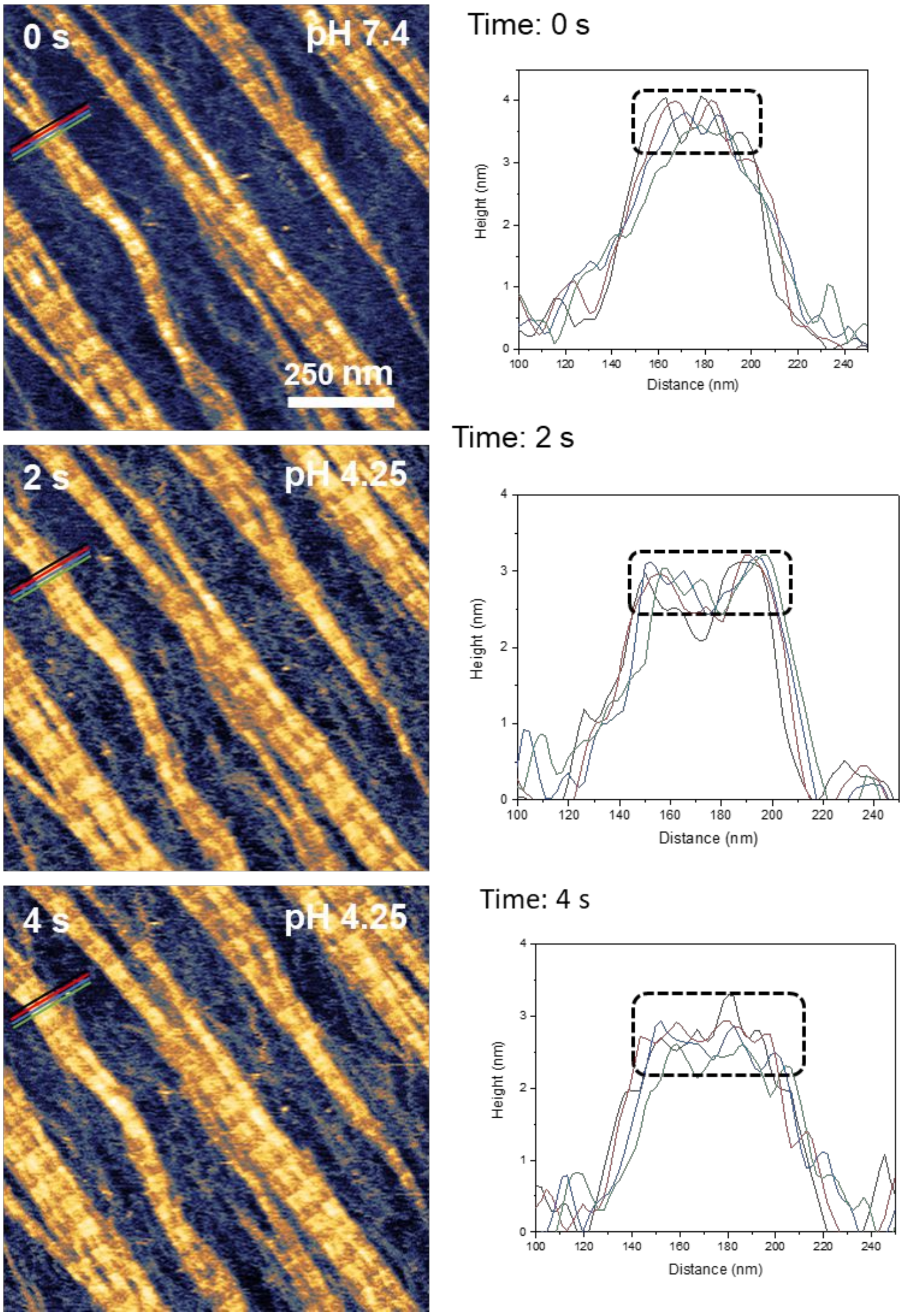

Supplementary Figure 4. Representative examples of the frames and height cross-sections used to determine the height evolution during disassembly presented in Figure 4. For each frame, the height values of four profiles (marked in black, red, blue and green). The average only included height values within the marked region. The HS-AFM images were obtained by applying a peak force of 610 pN Imaging rate, 1 fps (512x256 pixels). Additional HS-AFM data, f_1 = 528 kHz, k_1 = 0.15 nN/nm, Q_1 = 1.4; A_0 = 3.5 nm, A_{sp} = 2.1 nm.

Molecular dynamics simulation

For our coarse-grained molecular dynamics simulations, we rely on the mimetic collagen model established in ref. 40. The disassembly-reassembly process is simulated through a series of simulations. First, we simulate a pre-assembled nanoribbon (consisting of 324 mimetic molecules) in contact with an implicit water bath at room temperature (T = 300K) until time $t_0 = 0\tau_0$, where τ_0 is the MD unit of time. At this point, we stop the simulation and in order to mimic a drop in pH, we remove the negatively charged residues at one end of the mimetic molecule and introduce additional positively charged residues at the other end (compare Figure 5 in main text). Additionally, we freeze the 55 innermost molecules around the backbone axis of the nanoribbon. The frozen molecules will not be able to move in the following simulation, however, they still interact with other molecules and in doing so, take on the role of the molecules that stay attached to the mica surface in the experiment. Then we continue with the simulation until $t_1 = 10\tau_0$. During this time, we observe the disassembly of the nanoribbon. To start the reassembly process, we again stop the simulation and reintroduce the original charge sequence of the mimetic molecule, which corresponds to increasing the pH back to neutral conditions. Following, we let the simulation run until $t_2 = 210\tau_0$, where we observe the reassembly of the nanoribbon in the early stages. After $30\tau_0$, the structure, its mass profile, and its FFT profile are already very similar to the initial situation at t_0.
